# Assessment of intertidal seaweed biomass based on RGB imagery

**DOI:** 10.1371/journal.pone.0263416

**Published:** 2022-02-24

**Authors:** Jianqu Chen, Xunmeng Li, Kai Wang, Shouyu Zhang, Jun Li, Mingbo Sun

**Affiliations:** 1 College of Ecology and Environment, Shanghai Ocean University, Shanghai, China; 2 Engineering Technology Research Center of Marine Ranching, Shanghai Ocean University, Shanghai, China; 3 East China Sea Environmental Monitoring Center, Shanghai, China; TDTU: Ton Duc Thang University, VIET NAM

## Abstract

The Above Ground Biomass (AGB) of seaweeds is the most fundamental ecological parameter as the material and energy basis of intertidal ecosystems. Therefore, there is a need to develop an efficient survey method that has less impact on the environment. With the advent of technology and the availability of popular filming devices such as smartphones and cameras, intertidal seaweed wet biomass can be surveyed by remote sensing using popular RGB imaging sensors. In this paper, 143 in situ sites of seaweed in the intertidal zone of GouQi Island, ShengSi County, Zhejiang Province, were sampled and biomass inversions were performed. The hyperspectral data of seaweed at different growth stages were analyzed, and it was found that the variation range was small (visible light range < 0.1). Through Principal Component Analysis (PCA), Most of the variance is explained in the first principal component, and the load allocated to the three kinds of seaweed is more than 90%. Through Pearson correlation analysis, 24 parameters of spectral features, 9 parameters of texture features (27 in total for the three RGB bands) and parameters of combined spectral and texture features of the images were selected for screening, and regression prediction was performed using two methods: Random Forest (RF), and Gradient Boosted Decision Tree (GBDT), combined with Pearson correlation coefficients. Compared with the other two models, GBDT has better fitting accuracy in the inversion of seaweed biomass, and the highest R^2^ was obtained when the top 17, 17 and 11 parameters with strong correlation were selected for the regression prediction by Pearson’s correlation coefficient for *Ulva australis*, *Sargassum thunbergii*, and *Sargassum fusiforme*, and the R^2^ for *Ulva australis* was 0.784, RMSE 156.129, MAE 50.691 and MAPE 28.201, the R^2^ for *Sargassum thunbergii* was 0.854, RMSE 790.487, MAE 327.108 and MAPE 19.039, and the R^2^ for *Sargassum fusiforme* was 0.808, RMSE 445.067 and MAPE 28.822. MAE was 180.172 and MAPE was 28.822. The study combines in situ survey with machine learning methods, which has the advantages of being popular, efficient and environmentally friendly, and can provide technical support for intertidal seaweed surveys.

## Introduction

In October 2018, the Intergovernmental Panel on Climate Change released a report calling on countries to take action to limit warming to 1.5°C. China has also set the goal of "peaking CO2 emissions by 2030 and becoming carbon neutral by 2060". China’s coastline is long and complex, with many seaweeds in the intertidal zone, and these seaweeds are a major component of the nearshore carbon pool. In addition, the degradation of intertidal seaweed is serious due to global warming and increasing ocean acidification caused by human activities and climate change. The survey of intertidal seaweed biomass by satellite and Unmanned Aerospace Vehicle (UAV) needs to be combined with in-situ sampling, which is the most laborious part of the survey, and on the other hand, large-scale sample collection will inevitably damage the nearshore ecological environment.

With the development of science and technology, the traditional biomass survey method, from quadrat survey gradually to remote sensing drones, satellite remote sensing and other efficient survey methods, the application of satellite for estimation can get the biomass results of the target species in a large range, but its accuracy is difficult to guarantee [1–3]. Yuhan Zheng et al. [4]. estimated the macroalgae farming area. With the popularity of drones, the method of inversion of biomass based on spectral information combines the advantages of a wide survey area and high accuracy, and is widely used in pastoralism and agriculture. Izar Sinde Gonzalez et al. [5]. retrieved the aboveground biomass AGB of forage through multi-source remote sensing data, and R^2^ was 0.78. The data obtained by the drone has a higher resolution, not only the spectral information of the measurement area can be obtained, but also the texture information can be obtained. Yinuo Liu et al. fitted the biomass of oilseed lettuce through the spectral and texture characteristics of UAV [6]. H. Z. et al. effectively estimated the aboveground biomass of rice in combination with the spectral and texture characteristics of UAV [7]. R^2^ in the whole growth stage was 0.75 and the highest at heading stage was 0.84. Numerous studies have shown that by combining spectral and texture features, UAV images have great potential in biomass assessment.

Normally, experimental investigators are complicated to operate and also need to be combined with the biomass data collected in situ for processing. Therefore, it is particularly important to carry out quick in-situ surveys. With the development of sensors, high-resolution RGB images have become readily available. Although common smart phones and cameras have lower spectral resolution than Multi-spectral and Hyper-spectral sensors, their spatial resolution is high enough to effectively extract texture features in the image. The gray level co-occurrence matrix proposed by Haralik R M et al. can effectively extract image texture information [8]. Huang X et al. studied multi-scale texture, shape feature extraction and object-oriented classification of high-resolution remote sensing images [9]. Sarker et al. have extracted the texture features of the image through the gray level co-occurrence matrix to inverse the forest biomass [10]. Therefore, through the texture features and spectral features of RGB images, an inversion model with biomass can be established to evaluate the in-situ biomass of seaweed in the intertidal zone. The method of inverting biomass through RGB images can only be carried out in a small area, but it can quickly and efficiently obtain above-ground biomass (AGB). The inversion of biomass through RGB images can be matched and applied with UAV images to form an integrated above-ground biomass Quantity evaluation survey system.

The application of remote sensing images to retrieve biomass is actually a regression problem between image parameters and biomass. At present, agricultural researchers commonly use logistic regression and simple linear regression for regression problems (Biplab Brahma) [11]. The fitting accuracy results are often unsatisfactory. With the popularity of machine learning, more and more studies in the field of remote sensing have begun to apply machine learning methods to regression problems. Amir Safari et al. retrieved Forest Aboveground biomass through Landsat 5 TM and lidar data and machine learning methods such as random forest and decision tree [12].

The machine learning method has good universality and robustness, and can train the model according to the input data set, so as to achieve the ideal regression accuracy, it is widely used in the Internet, medical, financial and other fields [13–15]. Common machine learning methods used for regression prediction include Random Forest (RF), Gradient Boosting Decision Tree (GBDT), etc. Therefore, in this paper, we will shoot with a 12-megapixel RGB sensor, extract 51 feature parameters of the photo, and apply two commonly used models, RF and GBDT, to perform parameter search and obtain the best parameters and the best accuracy of the model. In addition, to avoid “dimensional disasters” [16, 17] caused by too many features, the input features are filtered by Pearson’s correlation coefficient to find the best inversion of the model.

## Materials and methods

Sample Collection and Preparation In situ quadrat photography of intertidal seaweeds from GouQi Island at low tide from 4 to 10 June 2021, 11:00–13:00 (UTC/GMT+08:00) in clear weather (Table 1) A quadrat of size 0.25m x 0.25m was used for collection according to the Marine Survey Specification [18, 19] (S1 Appendix). Each intertidal survey section was divided into three stations, one for high tide, one for mid tide and one for low tide, and a total of nine quadrats (3 x 3 parallel samples) were collected from each sampling station (S2 Appendix), according to the method of Stphenson and Vaillant for the division of tidal zones and stations [20]. This experiment was shot with a sensor resolution of 12 megapixels (iPhone 7plus) and at a height of 0.5m above each quadrat. After filming, latitude and longitude were recorded and all target species in the quadrat were collected, drained and brought back to the laboratory for biomass determination (S3 Appendix). The target species for this study were the dominant species commonly found in the intertidal zone of GouQi Island: *Ulva australis*, *Sargassum thunbergii*, and *Sargassum fusiforme*.

**Table 1:** Sample sites.

Longitude / °	Latitude / °
122.778	30.731
122.772	30.725
122.760	30.719
122.752	30.710
122.757	30.704
122.768	30.709
122.773	30.705
122.776	30.698
122.785	30.702
122.783	30.706
122.781	30.711
122.779	30.714
122.785	30.721
122.789	30.722
122.791	30.721
122.794	30.722

Since RGB data is collected only in one season, in order to further illustrate the repeatability of this paper, we used ASD Field Spec Handheld (Field Spec Handheld, ASD, USA) to measure the hyperspectral data at each growth stage of seaweed. The seaweed in the intertidal zone of GouQi island are in the prosperous period in summer and autumn and in the declining period in winter and spring (S4 Appendix). Therefore, we conducted hyperspectral measurements on seaweed of different lengths and sizes (different growth stages) in the sunny and cloudless noon 11:30–13:30 (UTC/GMT+08:00) in autumn and winter (October 17–24, 2019; January 1–7, 2020) [21, 22].

### Data analysis

For the problem of regression between image parameters and biomass, common machine learning methods used for regression prediction include Random Forest, Gradient Boosting Decision Tree, etc. [23]. As a non-parametric learning algorithm, the random forest model is integrated by multiple decision trees, and the model is simple and fast to build. It can effectively solve the problems of covariance and overfitting caused by the large amount of data, and can be used to solve regression and classification problems. Support vector machine models for regression prediction, where the computation and computational complexity of the hyperplane depends on a small number of support vectors rather than the entire sample space, are widely used in regression and classification problems [12]. While GBDT regression prediction, GBDT (Gradient Boosting Decision Tree) also known as MART (Multiple Additive Regression Tree), is an iterative decision tree algorithm, which consists of multiple decision trees and the conclusions of all the trees are accumulated to make the final answer. It was considered a more generalizable algorithm along with SVM when it was first proposed, but has less application in remote sensing [24].

As the quadrat collected are less likely to have all three seaweeds present at the same time, the biomass of the third seaweed is 0 when there is coexistence of two seaweeds. Therefore, when the third seaweed is selected, a biomass of 0 is eliminated, so the sample sizes of the three seaweed are not identical. These photos (S1 Appendix) are used to extract spectral information and texture information after calculating their GLCM.

Yinuo Liu uses 4 spectral parameters and 10 texture parameters for regression modeling of winter rape (*Brassica napus* L.) biomass [6]. In order to explore the optimal number of parameters used in regression modeling, each in-situ photograph in this article calculates the vegetation index by the spectral band (24 spectral features), Gray-level co-occurrence matrix of each image (Gray-level co-occurrence matrix, GLCM), Then extract the texture feature parameters to get 27 texture features, 51 features in total. It is equivalent to converting each RGB image into a one-dimensional array consisting of 51 parameters and a biomass label. RF and GBDT are used for regression modeling respectively, and 51 parameters are screened through Pearson correlation coefficient to obtain the best number of input parameters. The specific spectral features and texture features are shown in Tables 2 and 3.

**Table 2:** Spectral characteristic parameters.

Vegetation Index	Formula
R	Average DN value of red band
G	Average DN value of green band
B	Average DN value of blue band
r	R/ (R+G+B)
g	G/ (R+G+B)
b	B/ (R+G+B)
Exg	2×g-b-r
NGBVI	(g-b) / (g+b)
GBRI	g/b
VEGI	g/ ((r^0.67^) ×b^0.33^)
NPCI	(r-b) / (r+b)
RGBVI	(g2 − (b× r)) / (g2 + (b ×r))
RGRI	r / g
RGMPI	r × g
RBRI	r / b
RBMPI	r × b
RBMI	r − b
RGMI	r − g
RGPI	r + g
RBPI	r + b
GBPI	g + b
GBMI	g − b
VDVI	(2×g − r − b) / (2×g+r+b)
VARI	(g − r) / (g + r − b)

**Table 3:** Texture feature parameters (red band).

Texture Parameters	Formula
Mean_R	[∑_*i*_ ∑_*j*_ *P*(*i*, *j*)]/N
Variance_R	$\left\{\left[\underset{i}{\sum }\underset{j}{\sum }{\left[P\left(i,j\right)-\bar{P}\left(i,j\right)\right]}^{2}\right]/\text{N}\right\}$
Homogeneity_R	$\underset{i,j}{\sum }\frac{p\left(i,j\right)}{1+\left|i-j\right|}$
Contrast_R	∑_*i*_ ∑_*j*_ *P*(*i*, *j*) (*i* − *j*)^2^
Second Moment_R	*∫−∞∞*(x*−*c)^*2*^f(x)dx
Entropy_R	∑_*i*_ ∑_*j*_ *P*(*i*, *j*) × (−*lgP*(*i*, *j*))
Correlation_R	[∑_*i*_ ∑_*j*_ ((*ij*)(*i*, *j*)) − μ_x_μ_y_]/σ_x_σ_y_
ASM_R	∑_*i*_ ∑_*j*_ *P*(*i*, *j*)^2^
IDM_R	∑_*i*_ ∑_*j*_ {*P*(*i*, *j*)/[1 + (*i* − *j*)^2^]}

Note: Since the GLCM calculated based on the original image is calculated in Table 3, mean_ R is different from the value of R in Table 3. μ_x_, μ_y_ is the mean, σx, σy is the variance. IDM means Inverse Different Moment. ASM means Angular Second Moment.

The 24 vegetation parameters are calculated based on three bands of RGB image, so the corresponding texture feature parameters are used for each band. In Table 3, the red band (_ R) For example, green band (_ G) And blue wave band (_ B) Similarly, there are 27 texture feature parameters.

In image texture analysis, the selection of calculation window size is very important. Too small a sliding window will lead to differences in the window, while too large a window will not be able to effectively extract texture information. Through comparative analysis, this paper finally selects the sliding window of 5 × 5 is analyzed.

In addition, this paper uses 51 features, which may lead to “dimension disaster” due to too many feature [16, 17]. Therefore, this paper adopts the machine learning feature parameter screening algorithm combined with Pearson correlation coefficient, and carries out exclusion screening from small to large through the absolute value of Pearson correlation coefficient, so as to obtain different regression results.

Due to the influence of water vapor absorption and other factors, the data before 400nm and after 900nm in the spectral data are noisy, so they are eliminated [25]. The measured data were averaged every 10, and the spectral curves of different stages of each seaweed were obtained [21, 22]. Quantitative analysis of spectral data by PCA.

## Result

### Spectral data analysis of seaweed

**Fig 1:**
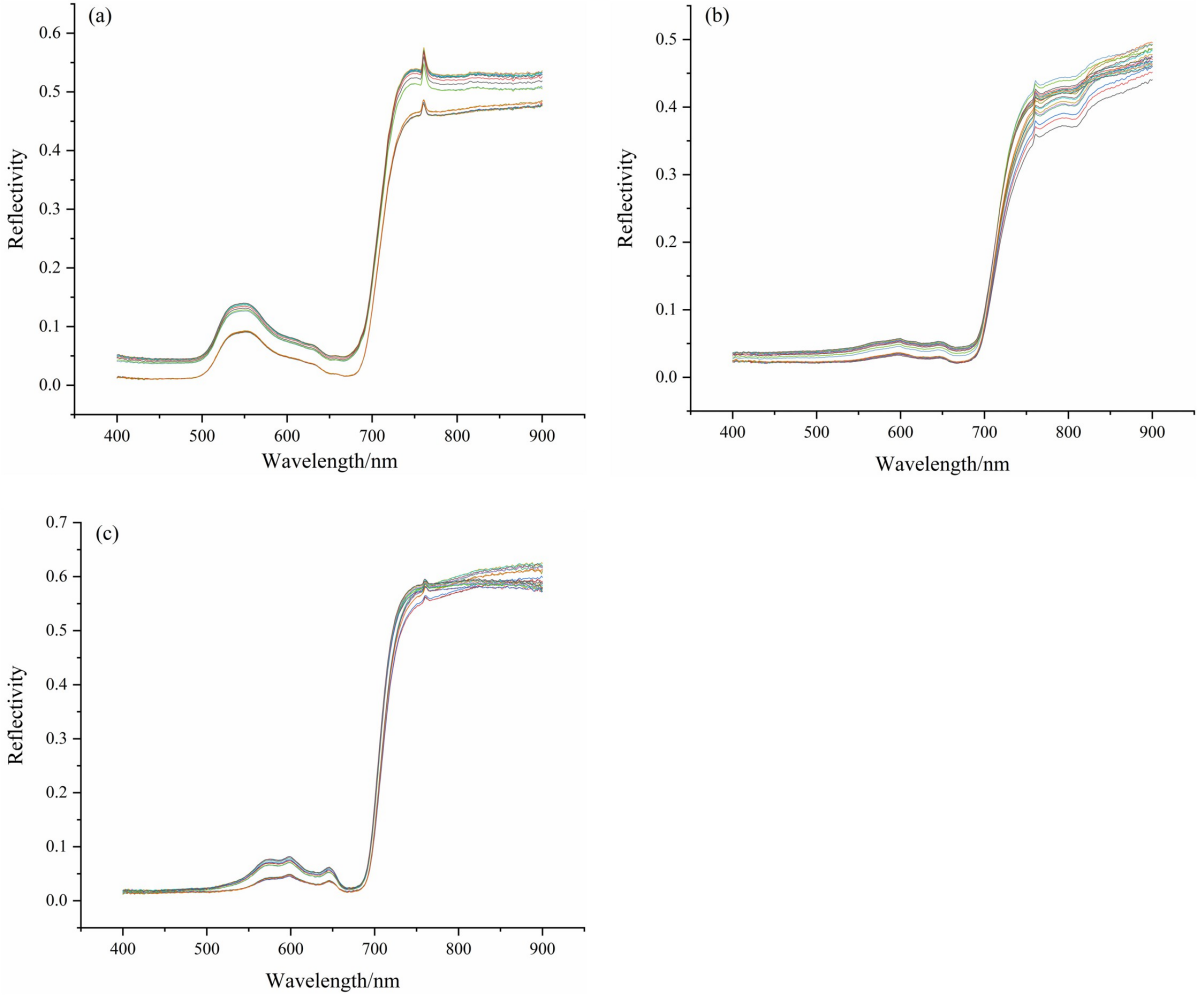
Spectral curves of seaweed at different growth stages. a. *Ulva australis*, b. *Sargassum thunbergii*, c. *Sargassum fusiforme*. The spectral curves of different colors represent the average of each growth stage.

The hyperspectral data of three seaweed species in the intertidal zone were measured by spectrometer, and the spectral curves of three seaweeds at different growth stages were obtained after data processing (Fig 1). Different color curves represent different stations and seasons. Through Fig 1, it can be intuitively and qualitatively concluded that there is little difference in spectral data at different growth stages of seaweed.

Through Principal Component Analysis (PCA), Most of the variance is explained in the first principal component, and the load allocated to the three kinds of seaweed is very high, all reaching more than 90%. It shows that the spectral curves of the three seaweed have limited changes in the data set. In this paper, RGB images only use these three bands of seaweed as input parameters, and do not use other bands. When the spectral is used to calculate vegetation index, normalization is performed. Therefore, this paper has repeatability and simple practical operation.

### Pearson correlation coefficient between parameters and biomass

**Fig 2:**
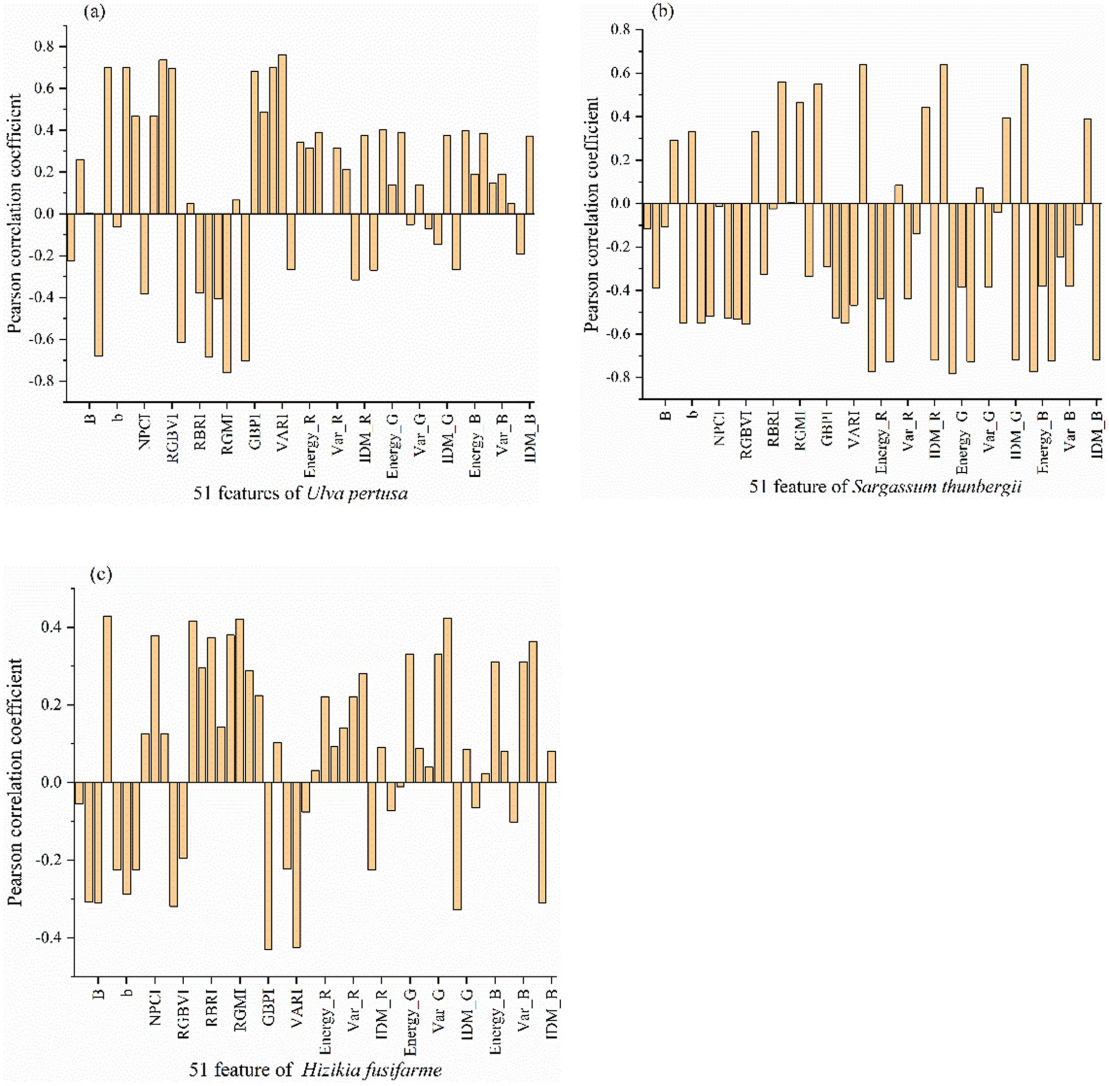
Pearson correlation coefficients of different parameters. a. Pearson correlation coefficient between 51 characteristics and biomass of *Ulva australis*, b. Pearson correlation coefficient between 51 characteristics and biomass of *Sargassum thunbergii*, c. Pearson correlation coefficient between 51 characteristics and biomass of *Sargassum fusiforme*.

Pearson correlation analysis was carried out for each characteristic parameter and biomass of RGB image, and Pearson correlation coefficients between 3 kinds of seaweed and 51 characteristic parameters and their respective biomass were obtained (Fig 2).

In Fig 1, the correlation coefficients between different spectral parameters, texture parameters and biomass vary greatly. There are about 17 characteristics with good correlation for each kind of seaweed. According to Pearson correlation coefficient, 51 parameters of three seaweeds were screened and preliminarily predicted by machine learning method. The correlation coefficients between 51 parameters and biomass are sorted from large to small according to the absolute values of these coefficients. Then, all these parameters are substituted into the model as dependent variables, and the results of 51 parameters are obtained. the parameters ranking last (the absolute value of correlation coefficient is the smallest) are removed, and 50 parameters are substituted into the model to obtain the results of 50 parameters. In this way, the parameters are eliminated one by one, and the operation results of different numbers of parameters are obtained. Until the last parameter. The change of prediction accuracy with the decrease of parameters is obtained. Among them, the correlation between the biomass of *Ulva australis* and the spectral characteristic parameters is higher, and the joint prediction R^2^ is higher than that predicted only by the spectral characteristic parameters or only by the texture features (R^2^ of RGBVI = 0.741, R^2^ of texture = 0.717, R2 of texture_ RGB = 0.784). In the regression between biomass and spectral characteristics of *Sargassum thunbergii*, it has a higher correlation with texture characteristic parameters, but compared with that of *Ulva australis*, *Sargassum thunbergii* has a better correlation with most characteristic parameters. Similarly, joint prediction is the best (R^2^ of RGBVI = 0.57, R^2^ of texture = 0.74, R^2^ of texture_ RGB = 0.854). *Sargassum fusiforme* is similar to *Sargassum thunbergii*, and there is little difference in the correlation with spectrum and texture, but the correlation between all characteristics and biomass is almost smaller than that of the other two seaweeds (R^2^ of RGBVI = 0.738, R^2^ of texture = 0.638, R^2^ of texture_ RGB = 0.808). Therefore, it is not that the more features, the better the regression effect. When there are too many features, it will lead to "dimension disaster" and reduce the prediction accuracy. Therefore, in order to improve the accuracy of estimation, parameter optimization is needed. In this paper, parameter screening is carried out by combining Pearson correlation coefficient. According to the absolute value of Pearson correlation coefficient, the R^2^ of biomass estimated by the model is obtained.

In the process of regression analysis, the R^2^ estimated by GBDT is the highest among the two models, so GBDT model can be selected for parameter screening in practical application. With the decrease of input parameters, R^2^ of biomass estimation shows different trends. The accuracy of joint parameters is higher than that of spectral parameters or texture parameters alone. Therefore, 51 parameters are screened, and the optimal parameters of *Ulva australis*, *Sargassum thunbergii* and *Sargassum fusiforme* were 17, 17 and 11 respectively (precision in Table 4). The same method was used to screen the parameters of the three seaweed in RF, and the optimal number of parameters was 15, 14, 10 respectively. The precision of RF is not as high as that of GBDT (Table 5).

**Table 4:** Three kinds of seaweed statistical parameters (GBDT).

seaweed	*Ulva australis*		*Sargassum thunbergii*		*Sargassum fusiforme*	
	Train set	Test set	Train set	Test set	Train set	Test set
R^2^	0.998	0.784	0.999	0.854	0.999	0.808
RMSE	13.550	156.129	20.559	790.487	18.608	445.067
MAE	10.420	50.691	16.828	327.108	15.341	180.172
MAPE	9.431	28.201	8.973	19.039	8.088	28.822

**Table 5:** Three kinds of seaweed statistical parameters (RF).

seaweed	*Ulva australis*		*Sargassum thunbergii*		*Sargassum fusiforme*	
	Train set	Test set	Train set	Test set	Train set	Test set
R^2^	0.931	0.759	0.919	0.781	0.897	0.719
RMSE	85.157	164.314	586.991	955.362	305.523	494.707
MAE	60.756	85.697	462.728	668.785	258.921	357.712
MAPE	34.695	557.985	271.383	2511.657	195.712	1694.078

### Comparison between predicted biomass and actual biomass

After dimension reduction by using the parameter screening method based on Pearson correlation coefficient, the optimal number of input parameters of each seaweed is obtained. These parameters are taken as feature, and the biomass of the three seaweeds are taken as labels. Two regression models of RF and GBDT are used for regression prediction, and the training set and test set are randomly divided according to the ratio of 3:1. The effective samples of *Ulva australis*, *Sargassum thunbergii* and *Sargassum fusiforme* were 100, 59 and 58 respectively.

In order to explore the best regression model, the parameters of each model are optimized. Through the Grid-Search Method (CV = 4), the optimal input parameters of each model are found, and different prediction graphs are obtained.

GBDT modeling is carried out in Python 3.8 environment through pycharm2020.1, and GBDT regression model is established by calling gradient boosting regressor in sklearn library. The traversal method is used to optimize the model parameters, in which the maximum iterations of the weak learner range from 100 to 1000, the step size is 10, and the weight of each weak learner is learning_ The rate ranges from 0.1 to 1 in steps of 0.05. After traversal, the best estimators and learning are obtained_ The rates were 100 and 0.1 respectively, and the parameters were used for regression prediction of the three seaweed.

In order to evaluate the accuracy of the model, we call r2_score from the sklearn library to calculate the R^2^ of the training set and the test set. Call numpy library to calculate RMSE and MSE. The training set of *Ulva australis*, *Sargassum thunbergii* and *Sargassum fusiforme* was obtained, and the highest R^2^, RMSE and MAE in the test set (Table 4).

In the GBDT model, after optimizing the input characteristics and model parameters by Pearson correlation coefficient and grid search method, the prediction model R^2^ of *Ulva australis*, *Sargassum thunbergii* and *Sargassum fusiforme* is improved by 0.008, 0.015 and 0.018 respectively compared with the non-optimized model.

Carry out RF modeling in Python 3.8 environment through pycharm2020.1, and establish RF regression model by calling random forest regressor in sklearn library. Number of decision trees n_ The range of estimators is 1 to 200, the step size is 1, and the maximum tree depth is max_ The depth range is 1 to 20, the step size is 1, and the maximum characteristic number is max_ Features range from 0 to 1, and the step size is 0.1. After traversal, the best n is obtained_ estimators, max_ depth, max_ The features are 21, 12 and 1 respectively, and this parameter is used for regression prediction of three kinds of seaweed.

Calling r2_score from sklearn library to calculate the R^2^ of training set and test set. Call numpy library to calculate RMSE and MSE. The training set of *Ulva australis*, *Sargassum thunbergii* and *Sargassum fusiforme* was obtained. The highest R2, RMSE and MAE in the test set are shown in Table 5.

## Discussion

In this paper, the biomass is evaluated by RGB image combined with machine learning method, which has a good inversion accuracy, indicating the feasibility of using RGB image to evaluate the biomass of intertidal seaweed. RGB images are easy to obtain and can be obtained by popular means such as smart phones and cameras. This method can efficiently obtain ecological index in large-scale surveys or in-situ surveys required for assessment in combination with satellites and UAVs [26]. Future research can explore other seaweed species, improve the investigation system of seaweed biomass in the intertidal zone, effectively evaluate the biomass of various seaweed in the intertidal zone, reduce the ecological damage caused by large-scale investigation and reduce the manpower and material resources consumed in the investigation.

*Ulva australis*, look like green plants, has obvious color characteristics. It is easy to extract by spectrum, but it is difficult to extract the spectral index of *Sargassum thunbergii* and *Sargassum fusiforme*. Therefore, RGB images obviously have too few spectral bands during spectral analysis; The texture of *Ulva australis* is chaotic, and the correlation between texture parameters and biomass is small, which is similar to reefs, mussels and other substrates. Therefore, the texture parameters calculated by gray level co-occurrence matrix may be disturbed by other substrates; In addition, *Ulva australis* is often covered at low tide [27]. In this paper, biomass inversion is only carried out through RGB image, so there are inevitably covered seaweed and errors. However, due to the distribution of the three in the intertidal zone with characteristics of changes in seaweed species along the offshore direction, the area covered is usually small [28]. From the regression results, the errors caused by above factors have little impact on the evaluation results. The total seaweed biomass in the study area is about 4200g/m^2^, which is consistent with relevant studies [29].

This paper applies the machine learning input parameter screening method combined with Pearson correlation coefficient, which can effectively improve the regression accuracy and effectively avoid the over fitting phenomenon caused by too many features and noise. The algorithm of this method is clear, simple and easy to use, and suitable for practical application. In addition, the correlation between spectral parameters and texture parameters and biomass can be sorted respectively, and then the spectral and texture features can be eliminated together, which may further improve the prediction accuracy. In this paper, the same evaluation methods are used for biomass evaluation of texture parameters, but the accuracy is lower than that of joint regression, which is consistent with the relevant research results [30]. The normalized correlation of r-band is good, which is basically consistent with the research of related studies (forage, wheat, rice, etc.) [21, 31, 32], indicating that the red band and its derived indexes play an important role in plant biomass inversion [33].

Finally, according to the survey results, the dominant species of seaweed1 in the intertidal zone of GouQi island in autumn are *Ulva australis*, *Sargassum thunbergii* and *Sargassum fusiforme*, and their biomass is 411.060 g/m^2^, 2380.388 g/m^2^, 1411.486 g/m^2^ respectively.

## Conclusion

Combined with spectral and texture features, the accuracy of Biomass Assessment of intertidal seaweed (*Ulva australis*, *Sargassum thunbergii* and *Sargassum fusiforme*) is higher than that of using only a single feature. Through parameter optimization by Pearson correlation coefficient, the optimal number of input features is about 11–17.

Applying machine learning method to biomass regression can get high precision results. In this paper, RF and GBDT models are used to predict the biomass of *Ulva australis*, *Sargassum thunbergii* and *Sargassum fusiforme* respectively. It is found that the GBDT model has higher accuracy than the RF models.

In this paper, the in-situ RGB image is used for biomass inversion for the first time. It is obtained that the dominant seaweed species in the intertidal zone of GouQi island in June 2021 are *Ulva australis*, *Sargassum thunbergii* and *Sargassum fusiforme* respectively, and their average biomass is about 411.060 g/m^2^, 2380.388 g/m^2^, 1411.486 g/m^2^.

## Supporting information

S1 Appendix Field data and model input data (picture). (RAR)

S2 Appendix Sampling station setting. (RAR)

S3 Appendix Model input data (wet weight). (RAR)

S4 Appendix Original spectral data. (RAR)

S1 Data (RAR)
